# Special issue on software citation, indexing, and discoverability

**DOI:** 10.7717/peerj-cs.1951

**Published:** 2024-03-26

**Authors:** Daniel S. Katz, Neil P. Chue Hong

**Affiliations:** 1 National Center for Supercomputing Applications, Department of Computer Science, Department of Electrical and Computer Engineering, School of Information Sciences, University of Illinois at Urbana-Champaign, Urbana, Illinois, United States of America; 2 Edinburgh Parallel Computing Centre, University of Edinburgh, Edinburgh, United Kingdom; 3 Software Sustainability Institute, University of Edinburgh, Edinburgh, United Kingdom

**Keywords:** Software, Citation, Indexing, Discoverability, FAIR principles, Research software

## Abstract

Software plays a fundamental role in research as a tool, an output, or even as an object of study. This special issue on software citation, indexing, and discoverability brings together five papers examining different aspects of how the use of software is recorded and made available to others. It describes new work on datasets that enable large-scale analysis of the evolution of software usage and citation, that presents evidence of increased citation rates when software artifacts are released, that provides guidance for registries and repositories to support software citation and findability, and that shows there are still barriers to improving and formalising software citation and publication practice. As the use of software increases further, driven by modern research methods, addressing the barriers to software citation and discoverability will encourage greater sharing and reuse of software, in turn enabling research progress.

## Introduction

Software is increasingly essential to research. It can be viewed as both a tool to be recorded (for reproducibility) and cited (for credit) as a part of scholarly research works, as well as an output of research that can be used, reused, and further developed.

In 2021, PeerJ Computer Science staff invited us to propose a special issue, and because of our interest in the role of software in research, we chose to focus on Software Citation, Indexing, and Discoverability. This choice was also partially based on our co-leadership of the FORCE11 Software Citation Implementation Working Group (2023), which followed on a set of software citation principles published in 2016 (Smith, Katz & Niemeyer, 2016), working to move the community from having an idea of what could and should be done in this area to actually having a culture of citing software. We were (and are) also pursuing other activities both individually and collectively, generally aimed at increasing the sustainability of research software, such as the Software Sustainability Institute (SSI) (Crouch et al., 2013) and the US Research Software Sustainability Institute (URSSI) (Carver et al., 2018).

Ensuring that the use of software in research, particularly in publications, is effectively understood and recorded leads to challenges in how it is cited, indexed, and discovered. These include challenges relating to: software metadata; identifiers for software and their relationship to those of other research objects, and other software; the role of other stakeholders such as indexes, libraries and registries; fostering adoption; development of related tools; and the role of the FAIR principles in this space. The special issue was intended to focus on recent work addressing these challenges, particularly in the context of the FORCE11 working group.

Between 2017 and 2023, the FORCE11 group published a set of software citation implementation challenges (Katz et al., 2019), published checklists for (paper) authors (Chue Hong et al., 2019b) and (software) developers (Chue Hong et al., 2019a), published best practices for software repositories and registries (Task Force on Best Practices for Software Registries et al., 2020), published guidance for journals (JATS4R, 2021; Katz et al., 2021; Stall et al., 2023), and worked on metadata systems such as CodeMeta (Jones et al., 2017) and CITATION.cff (Druskat et al., 2021).

Additional relevant work in this area includes: FAIR for Research Software (FAIR4RS), which has created a new set of FAIR (findable, accessible, interoperable, and reusable) principles specifically for research software (Chue Hong et al., 2022); the work of the RDA/FORCE11 Software Source Code Identification working group to produce use cases for persistent identifiers for software source code (Research Data Alliance/FORCE11 Software Source Code Identification WG et al., 2020); the European Open Science Cloud’s Task Force report on Scholarly Infrastructures of Research Software (European Commission and Directorate–General for Research and Innovation, 2020); NISO’s efforts to standardize reproducibility badging, including metadata relevant for citation (NISO, 2021); and the work of the Digital Preservation Coalition and Software Preservation Network on motivations for preserving software (Morrissey, 2020).

The call for the special issue was issued in 2021, and in 2022, five papers successfully passed through the peer review and publication process. The remainder of this editorial discusses the papers and their potential impact, and where we think things are going next.

## Papers and impact

The final special issue contains the following published articles:

- Schindler et al. (2022): “The role of software in science: a knowledge graph-based analysis of software mentions in PubMed Central.” This work built a 300-million-triple knowledge graph of 11.8 million software mentions and affiliated metadata generated through supervised information extraction models that distinguish different types of software and mentions, trained on a gold standard *corpus*, and applied to more than three million scientific articles. It then used this graph to perform a large-scale analysis of software usage and citation practices. The analysis provides insights into the evolution of software usage and citation patterns across various fields, ranks of journals, and impact of publications. The authors publicly share all their data and models to facilitate further research into scientific use and citation of software.
- Frachtenberg (2022): “Research artifacts and citations in computer systems papers.” This paper studies the field of computer systems, which involves the engineering, implementation, and measurement of complex systems software and data. In this field, the reproducibility and replicability of research results depends on the availability of artifact, because system software often embodies numerous implicit assumptions and parameters that are not fully documented in articles. The work built a cross-sectional dataset of papers from 56 contemporaneous systems conferences, including data on conferences, papers, authors, citation counts, and the release, ongoing availability, badges, and locations of associated research artifacts. This data showed that artifacts were shared in 30% of all conference papers and 43% of papers in conferences that actively evaluated artifact sharing, and that the papers with shared artifacts had 75% more citations. Even after controlling for numerous confounding covariates, the release of an artifact increased a paper’s citations by 34%.
- Du et al. (2022): “Understanding progress in software citation: a study of software citation in the CORD-19 *corpus*.” This work investigated progress toward improved software citation by examining current software citation practices. It used a machine-learning-based data pipeline to extract software mentions from a collection of more than 280,000 scholarly articles on COVID-19 and related historical coronaviruses. The authors then closely examined a sample of the extracted software mentions and searched online for the mentioned software projects and their citation requests, in order to understand the status of software citation. Positively, they found increasing mentions of software versions, increasing open source practices, and improving software accessibility. However, they also found high numbers of informal mentions that didn’t credit software authors, as well as problems where software developers requested citations that did not match software citation advocacy recommendations and that were not followed by paper authors. Finally, they discussed implications for software citation advocacy and standard making efforts seeking to improve the situation.
- Garijo et al. (2022): “Nine best practices for research software registries and repositories.” Differing from the previous papers, this work was about the role of registries and repositories that aim to include software in their contents. These systems have a key role in supporting and improving software findability and research transparency, providing information for software citations, and fostering preservation of computational methods in a wide range of disciplines. However, developing them takes effort and there are few guidelines available to help their creators and operators. To address this need, the previously mentioned FORCE11 Software Citation Implementation Working Group (2023) convened a task force to distill the experiences of the managers of existing resources in setting expectations for all stakeholders. This paper described the resultant best practices, which include defining the scope, policies, and rules that govern individual registries and repositories, along with the background, examples, and collaborative work that went into their development. The paper’s authors believe that establishing specific policies such as those presented here will help other scientific software registries and repositories better serve their users and their disciplines.
- Cadwallader & Hrynaszkiewicz (2022): “A survey of researchers’ code sharing and code reuse practices, and assessment of interactive notebook prototypes.” While the first three papers studied research works, and the fourth studied research repositories and registries, this paper studied researchers themselves. It asked researchers in computational biology about how often and why they look at code (most often, to gain a better understanding of the article), which methods of accessing code they find useful (most often, links to a code repository containing an archived version of the software) and why, what aspects of code sharing are important to them (ensuring that the code was running in the correct environment and sharing code with good documentation), and how satisfied they are with their ability to complete these tasks (generally, they were satisfied). The paper also asked researchers to examine a specific code-sharing tool that would enable readers to easily run the code, and if they would be willing to spend more time to use this tool. The average researcher was found to be unwilling to incur the additional costs (in time, effort or expenditure) that are currently needed to use code sharing tools alongside a publication. Based on this, the authors determined that different models are needed for funding and producing interactive or executable research outputs if they are to reach a large number of researchers.

These papers both create a set of work that can be further developed to better understand software citation, indexing, and discovery, and also demonstrate factors that should lead to increased understanding of the role of software in research and the importance of the work of its developers and maintainers.

Schindler et al. (2022) helps us understand how software is used and cited through their analysis of the published record as captured in PubMed Central, which can be considered a baseline of data and tools that can be used to collect future data and understand future changes as well.

Frachtenberg (2022) looks at artifacts, including software, and finds that their presence, at least in computer systems conference papers, leads to increased citations of these papers, which hopefully will lead to increased sharing of such artifacts in the future. Reuse of these artifacts should lead to increased software sharing in this field, and better recognition of software efforts.

Du et al. (2022) overlaps with Schindler et al. (2022) and Frachtenberg (2022) to some extent, though using a somewhat different set of research papers. It also focused on formal recommendations for citation, and how both software developers follow them as well as how paper authors use the requests from software developers.

Garijo et al. (2022) discusses important practices for repositories and registries that store or refer to software. Having a set of such practices leads to more better citations, indexing, and discoverability of software as used in papers and other research works.

Cadwallader & Hrynaszkiewicz (2022) helps us understand how researchers use code associated with publications, how they share it statically today, and the difficulties in sharing it more dynamically in the future.

## Looking forward

The papers in this special issue clearly provide a snapshot of citation practices today, and they define needs in the field and the research ecosystem, including for:

- Better and ongoing collection of data about software citation in publications. In particular, we observe that two to three of the five papers took advantage of the fact that a high fraction of the biomedical literature has been openly available *via* PubMed. This leads to collected data that represents this field, but may not represent other fields as well, if the fields and their practices differ. As open access publications become more common across fields, we hope that other disciplines will become as well studied as biomedicine, and that any disciplinary differences will become apparent so that they can be addressed.
- Better communication about recommended software citation to software developers, leading them to make citation requests that are more likely to be followed by paper authors.
- Better citation practices by paper authors, perhaps following community/discipline-specific guidelines. Note that very recent work by Ram & Howison (2023) has found that practitioners often understand *how* to cite software but have widespread uncertainty about community norms on *which* software to cite, given the limited available space for references in papers, so it seems likely that this need involves both the how discovered by papers in this special issue as well as the which.
- More consistent use of best practices for registries and repositories that store or refer to software.
- Policies and tools that are very low cost (in time and money) for researchers to use to include their software in publications, if we want to move beyond links from publications to static software in repositories, towards more interactive or reusable forms.

The increasing use and ubiquity of software in research—now also driven by data science, machine learning, and open research/open access—emphasises the importance of software citation on transparency, reproducibility and reusability of research.

Addressing these needs both individually and collectively will lead to software that is more frequently cited, indexed, and discovered, encouraging more software sharing and reuse, and in turn leading to better and faster research progress.

## Additional Information and Declarations

### Data Availability

The following information was supplied regarding data availability: This is an Editorial.
